# Selected Area Deposition of High Purity Gold for Functional 3D Architectures

**DOI:** 10.3390/nano13040757

**Published:** 2023-02-17

**Authors:** John Lasseter, Philip D. Rack, Steven J. Randolph

**Affiliations:** 1 Department of Materials Science and Engineering, University of Tennessee, Knoxville, TN 37996, USA; 2 Center for Nanophase Materials Sciences, Oak Ridge National Laboratory, Oak Ridge, TN 37831, USA

**Keywords:** selected area deposition, chemical vapor deposition, laser heating, 3-dimensional nanostructures

## Abstract

Selected area deposition of high purity gold films onto nanoscale 3D architectures is highly desirable as gold is conductive, inert, plasmonically active, and can be functionalized with thiol chemistries, which are useful in many biological applications. Here, we show that high-purity gold coatings can be selectively grown with the Me_2_Au (acac) precursor onto nanoscale 3D architectures via a pulsed laser pyrolytic chemical vapor deposition process. The selected area of deposition is achieved due to the high thermal resistance of the nanoscale geometries. Focused electron beam induced deposits (FEBID) and carbon nanofibers are functionalized with gold coatings, and we demonstrate the effects that laser irradiance, pulse width, and precursor pressure have on the growth rate. Furthermore, we demonstrate selected area deposition with a feature-targeting resolutions of ~100 and 5 µm, using diode lasers coupled to a multimode (915 nm) and single mode (785 nm) fiber optic, respectively. The experimental results are rationalized via finite element thermal modeling.

## 1. Introduction

Three-dimensional printing of functional nanoscale materials and architectures is very challenging. To date, focused electron and ion beam-induced deposition is capable of high-fidelity and complex geometries, but the deposited material is typically contaminated by residual precursor by-products. While a significant amount of research has been invested in developing new precursors [1,2,3,4,5,6] and purification strategies [7,8,9,10,11,12,13,14], functional and pure 3D deposits remain a significant challenge. While research and applications of 3D printing of mesoscale and macroscale objects have surged in recent years, sub-µm 3D printing has been more limited. As recently reviewed by Hirt et al. [15], several techniques have been developed, such as direct ink writing, electrohydrodynamic printing or laser-assisted electrophoretic deposition, meniscus-confined electroplating, and laser-induced photoreduction. While each technique has relative strengths and weaknesses, most only approach the µm scale. Two photon lithography is capable of three-dimensional nanoprinting below the optical diffraction limit [16], but limited photoresist availability restricts the materials that can be accessed. Scanning electron and ion beam-induced deposition techniques, however, have recently been shown to be capable of 10 nm scale resolution with high-fidelity 3D printing. See Winkler et al. [17] for a recent review.

We recently demonstrated selected area deposition of PtC_x_ coatings on FEBID PtC_x_ deposits via a pulsed laser chemical vapor deposition (CVD) process [18]. The selected area deposition is a result of the constrained thermal transport that occurs in the pseudo-1-dimensional nanoscale architectures and thus deposits specifically on the FEBID structures with negligible growth on the substrate. The process was shown to be consistent with a pyrolytic CVD process where the precursor decomposition temperature is reached by controlling the laser parameters as well as the dimensions and thermophysical properties of the 3D FEBID template. The total growth is a function of the time that the nanostructure exceeds the dissociation temperature and the precursor flux on the nanostructure surface during the exceeded temperature. The concept was demonstrated using the well-known MeCpPt^IV^Me_3_ precursor and was consistent with thermal CVD results [19], where residual carbon remains in the coatings.

Given the limited functionality of the PtC_x_ material presented in our previous work, here we explored the dimethyl (acetylacetonate) gold (III) (Me_2_Au (acac)) precursor, as gold is conductive, biocompatible, resistant to oxidation, exhibits low plasmonic losses, and its surfaces can be chemically modified through processes such as thiolation. While nanoscale 3D applications are limited due to the FEBID purity described above, several example applications have emerged. For instance, magnetic 3D structures [20,21,22], vibrating sensors [23], chiral [24,25,26,27,28,29,30,31] and split ring resonators [32,33], and other [34,35] plasmonic devices, scanning probe tips [36,37,38], and some nanomechanical applications [39,40] have all been demonstrated.

Selected-area pyrolytic laser CVD of gold has been thoroughly studied by Baum et al. for circuit repair applications in the 1980s [41]. They showed pure pyrolytic CVD could be achieved with the Me_2_Au (acac) precursor when the laser-induced temperature rise from a focused laser exceeded the ~450 K decomposition temperature [42]. CVD studies with the Me_2_Au (acac) precursor are in good agreement with this decomposition temperature [43]. However, precursor decomposition as low as 363 K has been demonstrated with O_2_ and ozone co-reactants [44]. Here we show selected area deposition can be achieved using the Me_2_Au (acac) precursor and be extended to three dimensions with sub-diffraction limit resolution (pillar diameter << laser wavelength), while yielding high purity functional gold coatings of a variety of nanostructures. For instance, we demonstrate selected area growth on 3D FEBID structures and carbon nanofibers via localized pyrolytic decomposition of the Me_2_Au (acac) precursor.

In this work, various experimental geometries and materials, laser conditions (power density, pulse width, duty cycle, and laser wavelength), as well as precursor pressure are explored to rationalize the selected area deposition process. We also demonstrate the use of both multimode fiber optic delivery (λ = 915 nm, 100 µm beam diameter) and single mode fiber optic delivery (λ = 785 nm, ~5 µm beam diameter). COMSOL finite element thermal modeling is also performed to validate some of the experimental findings. We show that the deposition rate is proportional to the temperature and flux of precursor gas molecules that impinge on the nanostructure when the laser heated nanostructure exceeds the decomposition threshold. However, since we employ a pulsed laser system, the deposition rate can also be controlled by the laser duty cycle (product of the pulse width and the frequency). Additionally, deposition can be controlled by nanostructure geometry, and z-dimension deposition can specifically be manipulated by the laser energy. Chemical analysis confirms that high-purity functional coatings above 95 atomic percent (at.%) can be achieved, which far exceeds what FEBID alone can accomplish.

## 2. Methods

FEBID nanopillars are grown in an FEI (Thermo Fisher Scientific, Waltham, Massachusetts, USA) Nova 600 dual beam microscope using the typical scanning electron microscopy (SEM) conditions of 5 keV and 98 pA with a stationary beam to grow the vertical pillars. Dimethyl (acetylacetonate) gold (III), also known as (CH_3_)_2_Au (C_5_H_7_O_2_), was purchased from STREM (CAS 14951-50-9). Table 1 overviews the growth times and precursors pertinent to each figure.

Carbon nanofibers (CNF) were grown via a plasma-enhanced CVD process using either a patterned nickel catalyst or a dewetted nickel film [see Melechko et al. [45] for an overview]. These nanofibers are vertically aligned during growth by an applied electric field and are also known as “vertically aligned carbon nanofibers” (VACNF). To selectively coat the FEBID and carbon nanofibers, we used a WAVIKS Inc. (Dallas, TX, USA) optical delivery system mounted onto the FEI Nova 600 for in situ selected area growth directly onto the FEBID nanostructures. Two laser sources are used: (1) a 16 W 915 nm wavelength laser diode coupled to a multimode fiber optic with a 100 µm diameter spot size and thus a maximum irradiance of ~2.1 GW/m^2^; (2) a 220 mW 785 nm wavelength laser diode coupled to a single mode fiber with an approximately 10 µm diameter spot size and thus a maximum irradiance of ~2.8 GW/m^2^. The laser’s optical powers were externally measured. The lasers are modulated with a pulse width generator capable of ~10 ns-CW pulse widths and up to 2.5 MHz frequency. In-situ scanning electron microscopy (SEM) was performed during the selected area’s growth. Energy Dispersive X-ray Spectroscopy (EDS, EDX) measurements were performed in a Zeiss Merlin scanning electron microscope. EDS measurements were taken at 5 kV and 205 pA with P/B-ZAF corrections. The point spectra were 60 s collections with 3% dead time; the map was a 5 min collection with drift correction and 6.6 nm pixel spacing. In our EDS quantification, we observe measurable Ga from the focused ion beam milling and Si from the scattered electrons interacting with the substrate; thus, we ignore these contributions in the Au/C quantification. Table 2 presents a range of physical parameters used in this study.

**Figure 1:**
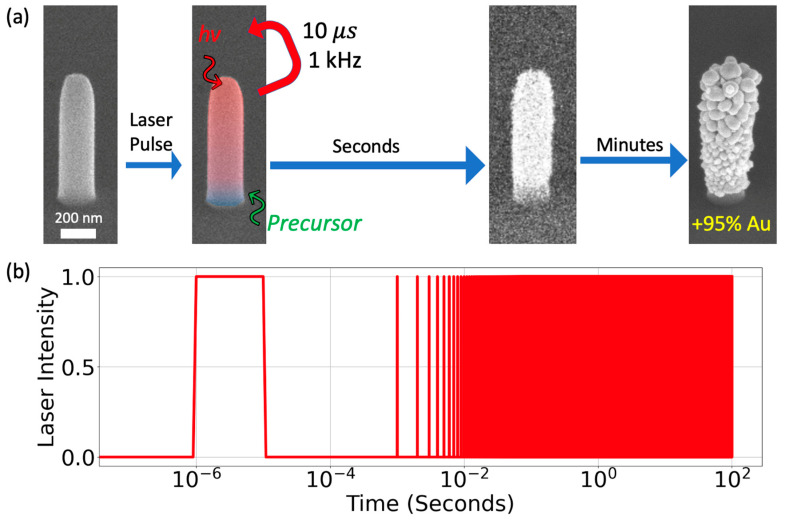
Schematic illustrating the temporal evolution during the selected area deposition process. (**a**) SEM images of an as-deposited FEBID nanopillar and subsequent images during the deposition process, where one is artificially colored to visualize the thermal effect of the laser. (**b**) A log time versus irradiance plot illustrating the various time scales that are operative where for instance the pulse width is 10 µs, the repetition rate is 1 kHz and the total processing time 200 s (with a 0.1% duty cycle and thus total laser on time of 0.2 s).

As illustrated in Figure 1, the CVD process is a pulsed process due to the pulsed laser source that selectively heats the nanostructures due to the unique thermal properties of nanoscale geometries. Pyrolytic CVD occurs for the duration in which the nanostructure temperature exceeds the dissociation temperature of the precursor. For sufficiently long pulse widths relative to the heating rise time, the growth rate is proportional to the laser on-time (product of the laser duty cycle and processing time) and the flux of the precursor molecules.

We focus here on low duty cycle pulsed growth, as, for instance, high irradiance continuous wave laser exposures can lead to overheating and failure in our optical delivery system. The low duty cycle ensures adequate cooling of the system during operation and allows us to perform in-situ imaging to discern the growth kinetics. Furthermore, we are able to control the growth at sub-monolayer pulse growth rates for superior control. While some nanostructure/substrate systems would in theory permit continuous wave growth, some combinations could lead to substrate heating at longer time constants and thus eliminate the selected area effect.

Finite-element modeling through the commercial COMSOL package was used to investigate the onset temperatures. The radiative beam is governed by the Beer-Lambert law in Formula (1):

(1) $$\frac{e_{i}}{⟦e_{i}⟧}*\nabla I_{i}=-\kappa I_{i}\;$$

where I is the radiative intensity, $e_{i}$ is the orientation of the ***i***th beam, and κ is the absorption coefficient. This model has three key assumptions: (1) The radiative beam in the absorbing medium is collimated, and each beam propagates always in the same direction, (2) the light experiences no refraction, reflection, or scattering within the material itself; and (3) there is no significant emission of the material in the wavelength range of the incident light. The radiative heat source $q_{r}$ corresponding to the energy deposited by the radiative beam is shown in formula (2):

(2) $$q_{r}=\sum_{i}\kappa I_{i}$$

The heat transfer in solids is modeled by the heat balance equation in formula (3):

(3) $$\rho C_{p}\left(\frac{\partial T}{\partial t}\right)+\nabla *\left(q+q_{r}\right)=Q$$

where ρ is the density, $C_{p}$ is the specific heat capacity at constant stress, T is the absolute temperature, q is the heat flux by conduction, $q_{r}$ is the heat flux by radiation, and Q contains additional heat sources.

COMSOL default silicon parameters were used: a specific heat of 700 $\frac{J}{kg*K}$, a density of 2329 $\frac{\mathrm{kg}}{m^{3}}$, and a thermal conductivity of 131 $\frac{W}{m*K}$. An absorption coefficient of 25,600 m^−1^ at 915 nm was used for silicon [46].

Previous literature values for PtC_5_ FEBID deposits [18] were used as starting points, and the nanostructure thermal conductivity was tuned until the pillar temperature reached the thermal CVD temperature of the Me_2_Au (acac), ~450 K. The optical parameters used at 915 nm for the nanopillars were an index of refraction of 1.87 and an extinction coefficient of 0.31 [47]. The laser was modeled as a flat top beam with a direction vector of $\left[\begin{array}{c} 0.419\\ 0.0588\\ -0.906 \end{array}\right]$ to mimic the laser angle during deposition. The nanopillar thermal parameters were: a specific heat of 700 $\frac{J}{kg*K}$ [48], a density of 1250 $\frac{\mathrm{kg}}{m^{3}}$ [48], and a thermal conductivity tuned to 1.05 $\frac{W}{m*K}$.

Carbon nanofibers were modeled as cones with experimentally determined height, base, and tip widths using COMSOL’s radiative beam in absorbing materials and heat transfer in solids as previously described. A single tip was used for modeling, but experimental geometries often have complex multi-tips. The graphite optical parameters used at 915 nm were an index of refraction of 2.2, and an extinction coefficient of 1.3 [49]. The nanopillar thermal parameters were a specific heat of 1000 $\frac{J}{\mathrm{kg}*K}$ [50], a density of 2200 $\frac{\mathrm{kg}}{m^{3}}$ [51], and a thermal conductivity tuned to 190 $\frac{W}{m*K}$. A tetrahedral mesh with a minimum element size of 10 nm was used on the nanopillars, and the nanofiber tetrahedral mesh’s minimum element size was 40 nm. Higher-resolution meshes were tested, but the same peak temperatures were reached and smoother gradients were the only effect.

## 3. Results and Discussions

**Figure 2:**
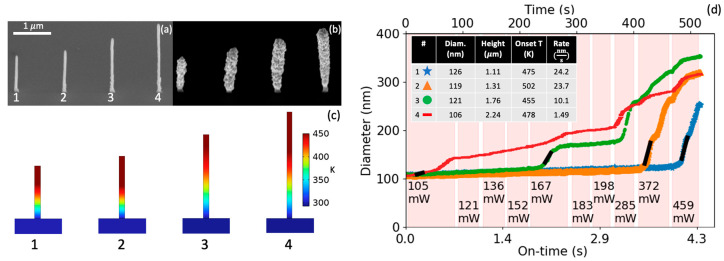
SEM images of the (**a**) as-deposited AuC_x_ nanopillars and (**b**) after the cumulative gold deposition realized during the laser power study. (**c**) Results from finite element modeling of the temperature profile at the end of the laser pulse width for the various nanopillars at the associated laser onset powers. (**d**) Plot of nanopillar diameter versus laser on-time (bottom *x*-axis) and processing time (top *x*-axis) with increasing laser power noted; the highlighted red regions correspond to the labeled laser powers. The inset table in (**d**) shows the nanopillar diameter, height, modeled onset temperature, and onset growth rate at the beginning of deposition.

Figure 2a is a scanning electron micrograph of the four as-deposited AuC_x_ (where x ~ 9) FEBID nanopillars grown from the Me_2_Au (acac) precursor that have comparable original diameters but various heights [see SEM movie capture in the Supplemental Information SI-1]. Figure 2d is a plot of the nanopillar diameter versus the laser on-time (the product of the processing time and duty cycle) and total processing time, where the laser power is incrementally increased over time from 105 to 459 mW (or 13.4–58.5 MW/m^2^). The laser is pulsed at a constant 1 kHz frequency with a 10 µs pulse width, and an image processing routine described in [18] is used to measure the average diameter of the top 67% of each nanopillar. Figure 2b is a scanning electron micrograph of the resultant gold-coated nanopillars after the experiment. To rationalize the onset temperature, we performed COMSOL finite element modeling of the peak temperature of the various nanopillar geometries and laser power for growth onset [see SI-2 for details], where the relevant properties used in the finite element model are listed. As illustrated in Figure 2c, all the onset temperatures exceed the ~450 K Me_2_Au (acac) thermal decomposition temperature. The inset in Figure 2d is a table listing the pillar diameter and height at the onset of growth, the modeled onset temperature, and the gold growth rate (normalized to the laser on-time) for each pillar (note the short black line on each plot denoting where the growth rate was taken). As expected, the laser power/irradiance that initiates growth is proportional to the FEBID nanopillar height; the taller pillars have higher thermal resistance, and thus the pillars exceed the Me_2_Au (acac) dissociation temperature at lower laser power. As illustrated in Figure 2c, the substrate heating is negligible, whereas significant heating is realized in the nanopillars, resulting in the realization of the selected area growth. As expected, the tips are the hottest, and the scanning electron micrographs after growth reveal a slightly tapered growth consistent with the thermal profile. Note that the high growth rate changes in the final three power intervals are due to larger increases in power. The slight long-term increase in the pillar diameter before selective deposition is attributed to electron beam induced deposition caused by the in situ secondary electron imaging of a FEBID precursor.

Consequently, once the gold layer is initiated, the optical and thermal properties change to a composite structure where the growing gold layer is optically in series with the AuC_x_ core material relative to the laser flux and is thermally in parallel relative to the substrate heat sink. While a concentric bi-layer model using gold surrounding an AuC_x_ core was explored, the optical absorption below ~50 nm is very sensitive to the surface roughness [52], which significantly enhances the optical absorption. Using standard gold properties has the effect of significantly reducing the structure temperature below the decomposition temperature, primarily due to significant reflectance but also due to enhanced thermal conduction (see SI-3 for an example and model details). Given that the deposition continues experimentally despite the predicted reflectance increase, we hypothesize that the observed surface roughness enhances the optical absorption, and thus growth is sustained at lower-than-expected optical powers. As shown in Figure 2d, the growth rates during a constant power interval can vary, which suggests temperature changes are attributed to the evolving optical and thermal properties of the composite structure.

**Figure 3:**
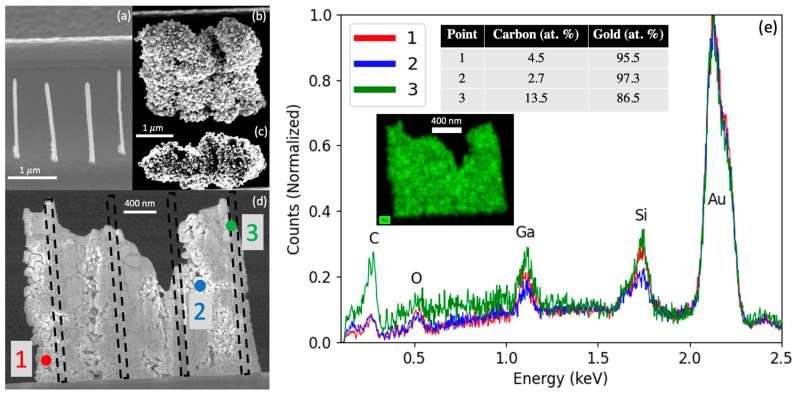
(**a**) A 52° tilted SEM image of the as-deposited PtC_x_ nanopillars. (**b**) A 52° tilted SEM of image of the nanopillars after gold selected area deposition. (**c**) Top-down SEM image of (**b**). (**d**) The Ga^+^ FIB thinned lamella and EDS measurement locations; the black dashed boxes estimate the positions of the original FEBID nanopillars. (**e**) The corresponding EDS spectra, taken at 5 keV 205 pA. The inset in (**e**) is the Au EDS map of the image in (**d**).

While EDS on individually grown nanopillars was attempted, inevitable scattering into the original PtC_x_ or AuC_x_ nanopillars made it difficult to distinguish the Au and C atomic compositions of the laser grown gold coatings. Figure 3a, illustrates a series of 4 PtC_X_ (where x ~ 5) nanopillars that were grown in close proximity and subsequently exposed to a pulsed laser at 10 µs pulse width, 1 k Hz frequency. The laser power was progressively ramped from 1.1 to 9.14 W (140–1164 MW/m^2^) over 10 min for the selected area growth (b) and (c) until all pillars had merged into a continuous wall. The nanopillar was a Ga+ focused ion beam milled into a thin lamella, as illustrated in Figure 3d, to measure the composition via EDS. Several point spectra are shown in Figure 3e, and inset is the associated Au EDS map of the entire lamella. By subtracting out the substrate Si and O peaks, points 1 and 2 reveal gold compositions of >95 atomic percent. Some variation in the carbon content as revealed in position 3 is evident (see C and Si maps in Supplemental Information SI-4); penetration of the electron beam and possible interaction with the FEBID core are suspected. Overall, the selected area of pulsed laser chemical vapor deposition from the Me_2_Au (acac) produces high purity Au nanostructured coatings.

**Figure 4:**
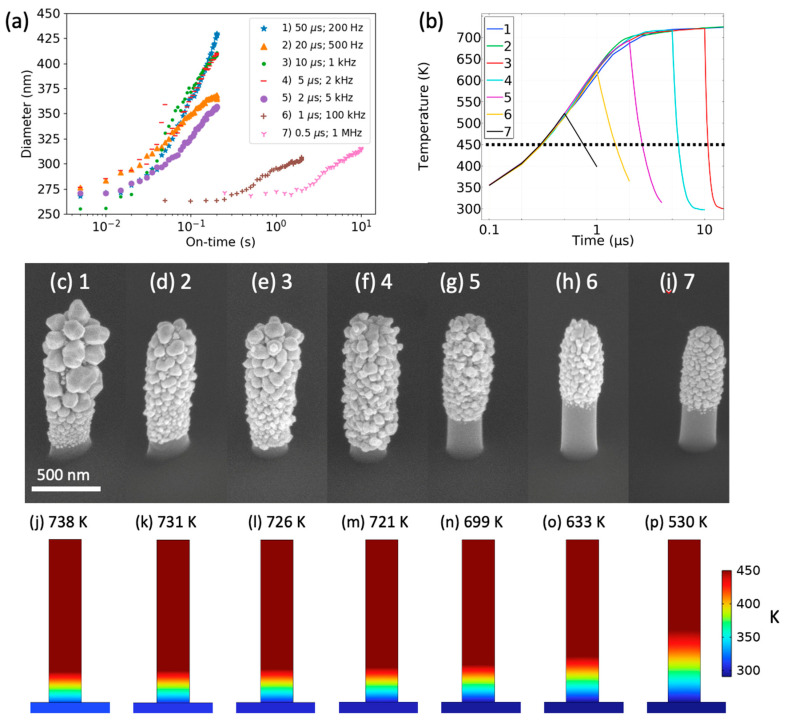
(**a**) A plot of the nanopillar diameters as a function of laser on-time at different pulse widths. (**b**) The simulated time versus temperature focused on 0.5, 1, 2, 5 and 10 µs pulse widths, as longer pulse widths have similar profiles to 10 µs. (**c**–**i**) SEM images of the nanopillar after processing at different pulse widths. (**j**–**p**) Modeled temperature profiles of the nanopillars at the end of the specified laser pulse width and peak temperature listed above the nanopillar.

In order to interrogate the Me_2_Au (acac) growth process in more detail, we performed a selected area growth study using PtC_X_ nanopillars grown via FEBID using the trimethyl (methylcyclopentadienyl)platinum (IV) precursor and varied the laser pulse duration and frequency at a constant irradiance (127 MW/m^2^). Pillars 1–5 are all grown at a constant duty cycle of 1%; pillar 6 is 10%; and pillar 7 is 50%. Figure 4a illustrates the average nanopillar diameter versus cumulative laser on-time for various laser pulse widths/frequencies, and Figure 4b is a plot of the modeled temperature versus time for each laser pulse width. Figure 4c–i are SEM images of the gold-coated nanopillars after selected area growth. At pulse widths of 5 µs and above, the growth rate is fairly consistent except for the 20 µs, 500 Hz growth, which has comparable early growth rates, but tapers off at ~6 × 10^−2^ s. Pillars 1–4 all have fairly uniform coatings that extend to the base of the nanopillars. The 2 µs pulse width gold thickness (~50 nm) is noticeably lower, and the coating only extends ~2/3 from the tip of the nanopillar. Finally, the 1 and 0.5 µs growths are indiscernible at 2 × 10^−1^ s, but do exhibit growth at 1 and 2 orders of magnitude longer laser on-times, respectively. As shown, these nanopillars exhibit a rise and decay time of ~2 µs. Of course, this time constant is related to the product of the thermal resistance and capacitance and is given by $\frac{\rho CpV}{kA}$, where ρ is the density, *C_p_* is the heat capacity at constant pressure, *V* is the volume, *k* is the thermal conductivity, and *A* is the surface area. As the pulse width is decreased, the rise time becomes a significant fraction of the pulse width, and thus the time above the dissociation temperature is a smaller fraction of the total laser on-time (see Figure 4b) for an illustrative line drawn at 450 K). At pulse widths lower than the rise time, the integrated time above the dissociation temperature diminishes, and thus much longer laser on-times are required for observable growth. Furthermore, the temperature evolution at short pulse widths limits the heating at the base of the nanopillars. Figure 4j–p illustrates the temperature profile of the nanopillars at the end of each pulse width, where the peak temperature is listed above the image. Interestingly, at the optimized 1 W/m-K thermal conductivity, the 5–50 µs pulse widths all have similar temperature profiles where a majority of the nanopillar exceeds the dissociation temperature. However, the 2, 1, and 0.5 µs pulse widths exhibit a progressively shorter region of the nanopillar tip region that exceeds the decomposition temperature. Thus, the growth rates and position-sensitive deposition as a function of pulse width consistently suggest the tuned thermal conductivity value is correct. One interesting feature that is also observed is that the grain structure in general becomes coarser with increasing pulse width.

**Figure 5:**
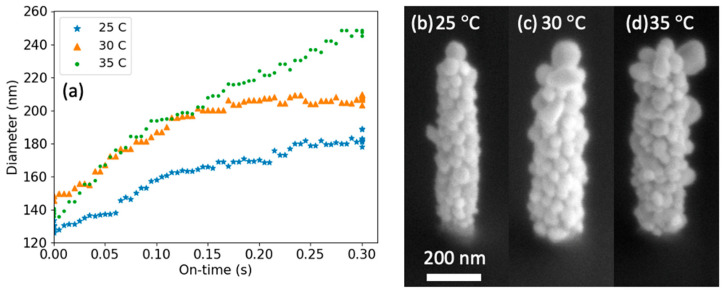
(**a**) A plot of the nanopillar diameters as a function of laser on-time at different crucible temperatures. (**b**–**d**) SEM images of the nanopillar after processing at (**b**) 25 °C, (**c**) 30 °C, and (**d**) 35 °C.

Next, we explored the effect of precursor pressure on the growth rate by comparing the growth on similar-geometry AuC_x_ FEBID nanopillars and varying the precursor crucible temperature (25, 30, and 35 °C) to raise the Me_2_Au (acac) vapor pressure/precursor flux during selected area deposition. For each crucible temperature, the same laser conditions of 10 µs, 1 kHz and 140 MW/m^2^ irradiance were used. Figure 5a compares a plot of the average nanopillar diameter as a function of laser on-time for the various crucible temperatures and clearly shows that the higher crucible temperature yields higher growth rates. Figure 5b–d are SEM images of the gold-coated AuC_x_ FEBID nanopillars grown at 25, 30, and 35 °C crucible temperatures, respectively; note that coarsening of the gold grains occurs at higher crucible temperatures. The average growth rates over the 0.3 s laser on-time are 92, 125, and 210 nm/s for the 25, 30, and 35 °C crucible temperatures, respectively. This agrees with our previous hypothesis [18] that the growth rate is controlled by the precursor flux and the time that the object’s temperature is greater than the precursor dissociation temperature.

**Figure 6:**
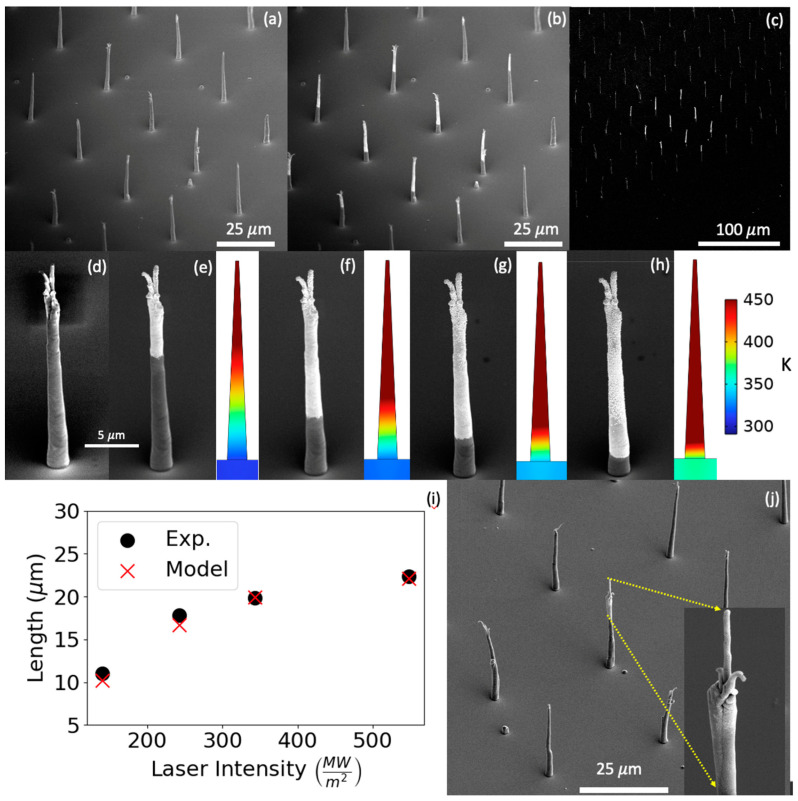
(**a**) SEM image of a CNF array before processing. (**b**) SEM image of array after laser processing. (**c**) The laser processed array at lower magnification, showing the area selectivity. (**d**–**h**) SEM images of a different CNF on the same sample, showing the spatial selectivity of the process as a function of laser power by exposing one fiber repeatedly while stepping the laser power at 915 nm 10 µs, 1 kHz; (**d**) as grown (**e**) 140, (**f**) 242, (**g**) 343 and (**h**) 548 MW/m^2^ irradiance. Insets next to (**d**–**h**) are temperature profiles of the carbon nanofiber exposed to the specific laser power illustrating the 450 K deposition temperature extends farther down the CNF at higher laser power. (**i**) An experiment vs. modeled plot of the laser power vs. deposition length, where the model is measured from the tip of the CNF to where the fiber temperature drops below 450 K. (**j**) SEM image demonstrating higher resolution selected area deposition using a laser coupled single mode laser diode with a spot size of ~10 µm diameter.

Finally, to test that the selected area deposition is a more generalized phenomenon that is compatible with structures other than FEBID-grown materials, we investigated functionalizing carbon nanofibers [53]. The CNFs were grown via a plasma enhanced CVD process using a lithographically patterned nickel catalyst [54,55]. Figure 6 shows an array of CNFs (a) prior to laser irradiation and (b) subsequent to a 20 s selected area gold deposition (343 MW/m^2^, 1 kHz, 10 µs), and (c) is a lower magnification image illustrating the approximate 100 µm laser spot size. To demonstrate *z*-axis selected area deposition control, Figure 6d shows an individual as-grown CNF, and post-growth images after a series of 30 s (300 ms laser on-time) selected area growths with 10 µs pulse width and 1 kHz frequency and (e) 140, (f) 242, (g) 343 and (h) 548 MW/m^2^ irradiance. Note in Figure 6d–h shows that the nanofiber has a secondary branch associated with the initial CNF. This is due to a stochastic splitting in the catalyst nanoparticle during the CNF plasma enhanced CVD process and is not associated with our selected area deposition process, which conformally coats the entire exposed fiber. Figure 6i illustrates that the length from the tip that is functionalized increases as a function of increasing power, and as expected, the gold thickness increases on the previously grown areas. We modeled this CNF geometry using optical and thermal parameters of graphite as described in the methods section, and note that the inset temperature maps of the nanofibers at each power show that the location along the CNF where the temperature exceeds the 450 K dissociation agrees well with the region of the selected area growth (for illustrative purposes, the temperature color is set to a maximum of 450 K—see Supplemental Information SI-5 for results where color scale is set to the maximum temperature located at the CNF tip).

While we did not perform continuous wave exposures to protect the optical delivery system, we performed a continuous wave simulation on the CNF system under the same 915 nm wavelength and 548 MW/m^2^ laser conditions. Under these conditions, substrate heating exceeds the precursor dissociation temperature, and we lose the desired site selectivity. See Supplemental Information SI-5 for modeling results of the temperature profile at the 0.3 s continuous wave total laser-on time.

In order to illustrate higher resolution selected area growth, we also tested a 785 nm wavelength laser diode coupled to a single mode optical fiber, which can yield comparable irradiance values at a spot size on the order of 5 µm [56]. The pulse width and frequency were 10 µs and 1 kHz, respectively, and the average laser irradiance was 598 MW/m^2^. Figure 6j illustrates that we can selectively target and functionalize a single CNF in this array with localization at the CNF tip.

## 4. Conclusions

A selected-area deposition of high-purity gold coatings on 3D nanostructures is demonstrated using the Me_2_Au (acac) precursor via a pulsed laser pyrolytic CVD process, which occurs due to the unique thermal properties of the nanoscale geometries. Good optical coupling and constrained thermal conduction allow selective heating of the nanostructures and negligible heating of the underlying substrates. Due to the fact that the nanopillar geometry controls the thermal conduction, the irradiance required to induce growth is proportional to the aspect ratio of the nanostructure. Above the irradiance onset value, where the laser pulse width is sufficiently longer than the thermal rise time, the growth rate is proportional to the laser on-time (i.e., the product of the duty cycle and processing time) and the flux of precursor gas that impinges on the surface during this time. For pulse widths shorter than the thermal rise time, the growth rate is decreased because the effective time that the nanostructure is greater than the precursor dissociation temperature is decreased. As the growth rate is proportional to the precursor flux and the time above the dissociation temperature at constant laser parameters, we show that the growth rate is proportional to the localized precursor pressure. We additionally validated selected area deposition as a more generalized nanostructural phenomenon by demonstrating the process on carbon nanofibers, where the z-dimension of gold functionalization is shown to be controllable via laser irradiance. We demonstrate both a 915 nm wavelength laser diode coupled to a multimode fiber optic capable of 100 µm spatial resolution and a 785 nm wavelength laser diode coupled to a single-mode fiber optic with 5 µm targeting resolution. Finite-element thermal simulations corroborate each of the experimental thermal studies. Chemical analysis of the gold coatings indicates purity in excess of 95 atomic percent, indicating the process is promising for producing functional gold coatings of nanostructured architectures.

## Figures and Tables

**Table 1:** Deposition Parameters.

Figure	Precursor Temperature (°C)	Template Material	Template Precursor	$\mathrm{Laser}\;\mathrm{Irradiance}\;\left(\frac{MW}{m^{2}}\right)$	Laser Wavelength (nm)	Laser on-Time (s)
1	30	PtC_x_	MeCpPt^IV^Me_3_	140	915	0.2
2	30	AuC_x_	Me_2_Au (acac)	13.4–58.5	915	4.3
3	30	PtC_x_	MeCpPt^IV^Me_3_	140–1164	915	5.5
4	30	PtC_x_	MeCpPt^IV^Me_3_	127	915	0.2–10
5	25, 30, 35	AuC_x_	Me_2_Au (acac)	140	915	0.3
6	30	CNF	C_2_H_2_ + NH_3_	598, 140–548	785, 915	0.3–1.2

**Table 2:** System Physical Parameters.

Parameter	Value
Laser	785 nm (single mode, r=5 μm), 915 nm (multi mode, r=50 μm)
Laser Intensity	13.4–1164 $\left(\frac{\mathrm{MW}}{m^{2}}\right)$
Laser Pulse Width	500 ns–50 μs
Laser Repetition Rate	200 Hz–1 MHz
FEBID SEM Voltage	5 kV
FEBID SEM Current	98 pA
Typical SEM Base Pressure	5 μTorr
SEM Room Temperature	25 °C
Typical Precursor Temperature	30 °C

## Data Availability

No publicly available datasets were used and all data created was original work. All data is available upon request but not publicly hosted due to large file size.

## Supplementary Materials

The following supporting information can be downloaded at: https://www.mdpi.com/article/10.3390/nano13040757/s1, SI-1. Figure 2 SEM Video of Me_2_Au (acac) CVD on Me_2_Au (acac) FEBID Nanopillars. Figure SI-2.1. Plot of temperature versus time for finite-element modeling of different thermal conductivities for parameter tuning. The modeled pillar is pillar 4 from main text Figure 2, 106 nm wide and 2.24 um tall with a 106 mW (13.5 MW/m^2^ irradiance) incident laser. Figure SI-3.1. The fitted thickness dependent thermal conductivity for thin Au films, from 1–250 nm. Figure SI-3.2. A time-temperature plot using the bilayer model with a gold thickness of 0 nm. Figure SI-3.3. A time-temperature plot using the bilayer model with 2, 5, 10, 30, 50 nm gold. Figure SI-4.1. An EDX map showing carbon concentration, with the cross-sectioned core FEBID pillar clearly visible in the bottom right. Figure SI-4.2. An EDX map showing silicon concentration, with the substrate showing high concentration and trace amounts on the pillar wall, likely from FIB cross-section re-deposition and beam scattering and interacting with the substrate. Figure SI-5.1. CNF thermal conductivity tuning, showing the temperature evolution over time at a given thermal conductivity value. Figure SI-5.2. Carbon nanofiber temperature profiles for various experimental powers of (a) 1.1 W, (b) 1.8 W, (c) 2.7 W, (d) 4.3 W, where the color scale bar is based on the peak temperature at the end of the laser pulse width. Figure SI-5.3. Modeled carbon nanofiber temperature profile of a 300 ms continuous-wave exposure, instead of the pulsed experiment and model in the maintext. References [18,57,58] are cited in Supplementary Materials.
