# Supplementary material for: Selected Area Deposition of High Purity Gold for Functional 3D Architectures
Source: Nanomaterials (Basel). 2023 Feb 17;13(4):757. doi: 10.3390/nano13040757 (PMC9965196; doi:10.3390/nano13040757)
Supplement: Supplementary file 1 [file nanomaterials-13-00757-s001.zip › nanomaterials-2172088-supplementary/supp/Selected Area Deposition of High Purity Gold for Functional 3D Architectures Supplement_Proofed.docx]

**Selected Area Deposition of High Purity Gold for Functional 3D Architectures Supplement**

*John Lasseter^1^, Philip D. Rack^1^, Steven J. Randolph^2,*^*

1. Department of Materials Science and Engineering, University of Tennessee, Knoxville, Tennessee 37996, United States

2. Center for Nanophase Materials Sciences, Oak Ridge National Laboratory, Oak Ridge, Tennessee 37831, United States

^*^Corresponding author email: randolphsj@ornl.gov

**SI-1 Figure 2 SEM Video of Me_2_Au(acac) CVD on Me_2_Au(acac) FEBID Nanopillars**

See attached files. A SEM video of selected area deposition on FEBID nanopillars via the laser-induced deposition process is shown (8x speed). This experiment is investigating the effect of laser irradiance, where all nanopillars are simultaneously exposed to the large 100 $\mu m$ 915 nm laser spot. The irradiance starts at 13.4 MW/m^2^ and is stepped up to 58.5 MW/m^2^ over time. The video shows the selectivity of the deposition process, as irradiance increases different geometries are pushed to the pyrolytic CVD temperature and begin deposition.

**SI-2** **Figure 2 Finite-Element Modeling of Me_2_Au(acac) FEBID Nanopillars Deposition Onset**

Previous literature values for PtC_5_ FEBID deposits^1^ were used as starting points, and the nanostructure thermal conductivity was tuned until the pillar temperature reached the thermal CVD temperature of the Me_2_Au(acac), ~450 K. The optical parameters used at 915 nm for the nanopillars were index of refraction of n=1.87, and an extinction coefficient of k=0.31. The laser was modeled as a flat top beam with a direction vector of $\left[ \begin{matrix} 0.419 \\ 0.0588 \\ -0.906 \end{matrix} \right]$ to mimic the laser angle during deposition. The nanopillar thermal parameters were: a specific heat (C_p_) of 700 $\frac{J}{kg*K}$, a density ($\rho$) of 1250 $\frac{kg}{m^{3}}$, and a thermal conductivity ($k$) tuned to 1.05 $\frac{W}{m*K}$.

**Figure SI-2.1.** Plot of temperature versus time for finite-element modeling of different thermal conductivities for parameter tuning. The modeled pillar is pillar 4 from main text Figure 2, 106 nm wide and 2.24 um tall with a 106 mW (13.5 MW/m^2^ irradiance) incident laser.

**SI-3** **Figure 2 Finite-Element Modeling of Me_2_Au(acac) FEBID Nanopillars with a Bilayer Au-FEBID Model**

Given that EDX results indicate a high-purity gold coating, we attempted to model the FEBID as deposited pillar and the conformal gold coating as a bi-layer thermal model. The laser radiation model described above is not compatible with a material bi-layer, thus, we used COMSOL’s heat transfer in solids and developed a bi-layer model based on power a modified heat source that we previously described^1^. In this model we use a simple series optical absorption model in which we have a vacuum/gold interface and a gold/PtC_x_ interface and reflection and absorption is determined at each interface and layer, respectively. For thin films below or close to the electron mean free path of 41 nm, the thermal conductivity is thickness dependent. The thickness dependent thermal conductivity equation was derived by fitting reference data^2^ to a power function: $58.656*D_{Au}^{0.2272}$ , where *D_Au_* is the gold thickness and is plotted in SI-3.1. Furthermore, reflectivity is also thickness dependent if the layer is below the absorption depth. The thickness dependent Au reflectivity equation was $0.44*(1-e^{-15.5*D_{Au}})$. A 915 nm 10 $\mu$s laser pulse for 0 nm of gold is modeled in SI-3.2, and 2, 5, 10, 30, 50 nm gold thicknesses are modeled in SI-3.3. Material parameters are shown in the table below. The finite element model predicts that the pillar temperature quickly drops below deposition temperature due to high gold reflectivity, but experimentally the deposition process proceeds, indicating that the gold properties differ from bulk values.

| PtCx Optical n | 1.87 |
| --- | --- |
| PtCx Optical k | 0.31 |
| PtCx Density | 1250 $\frac{kg}{m^{3}}$ |
| PtCx Thermal k | 1 $\frac{W}{m*K}$ |
| PtCx Specific Heat | 700 $\frac{J}{kg*K}$ |
| Au Optical n^3^ | 0.22 |
| Au Optical k^3^ | 6.17 |
| Au Density | 19300 $\frac{kg}{m^{3}}$ |
| Au Specific Heat | 128 $\frac{J}{kg*K}$ |

**Figure SI-3.1**. The fitted thickness dependent thermal conductivity for thin Au films, from 1-250 nm.

**Figure SI-3.2**. A time-temperature plot using the bilayer model with a gold thickness of 0 nm. Note that the maximum temperature here is slightly lower than is reported in maintext Figure 2, which is attributed to the usage of the simpler heat-dump model for the bilayer here vs. the radiative beam model used in maintext Figure 2 due to the heat module’s better compatibility with multiple layers.

**Figure SI-3.3**. A time-temperature plot using the bilayer model with 2, 5, 10, 30, 50 nm gold. A large drop in temperature is predicted which suggests the deposition would be halted soon after the gold incubation. Experimentally we do not observe this, thus we attribute the enhanced absorption to the film roughness.

**SI-4** **Figure 3 Full EDX Maps**

The full EDX maps of relevant species of the prepared sample in Figure 3. EDX maps of C (SI-4.1) and Si (SI-4.2).

**Figure SI-4.1**. An EDX map showing carbon concentration, with the cross-sectioned core FEBID pillar clearly visible in the bottom right.

**Figure SI-4.2**. An EDX map showing silicon concentration, with the substrate showing high concentration and trace amounts on the pillar wall, likely from FIB cross-section re-deposition and beam scattering and interacting with the substrate.

**SI-5** **CNF Finite-Element Modeling Parameters**

Carbon nanofibers were modeled as cones with experimentally determined height, base and tip widths using COMSOL’s radiative beam in absorbing materials and heat transfer in solids as previously described. A single tip was used for modeling, but experimental geometries often have complex multi-tips. Graphite optical parameters used at 915 nm were an index of refraction of n=2.2, and an extinction coefficient of k=1.3. The nanopillar thermal parameters were a specific heat (C_p_) of 1000 $\frac{J}{kg*K}$, a density ($\rho$) of 2200 $\frac{kg}{m^{3}}$, and a thermal conductivity ($k$) tuned to 190 $\frac{W}{m*K}$. Figure SI-5.1 shows a tuning plot of the temperature versus time for various CNF thermal conductivity values at the initial deposition power of 1.1 W (140 MW/m^2^), where the lowest power onset depth is tracked (11um). Figure SI-5.2 shows the nanofiber temperature distributions without highlighting of the deposition temperature. Figure SI-5.3 is a continuous wave modeled heat profile of the 548 MW/m^2^ 0.3 s on-time exposure in maintext figure 6, where the substrate itself can reach deposition temperature from CW exposures.

**Figure SI-5.1.** CNF thermal conductivity tuning, showing the temperature evolution over time at a given thermal conductivity value.

**Figure SI-5.2.** Carbon nanofiber temperature profiles for various experimental powers of (a) 1.1 W, (b) 1.8 W, (c) 2.7 W, (d) 4.3 W, where the color scale bar is based on the peak temperature at the end of the laser pulse width.

**Figure SI-5.3.** Modeled carbon nanofiber temperature profile of a 300 ms continuous-wave exposure, instead of the pulsed experiment and model in the maintext.

**Bibliography**

(1)	Lasseter, J.; Rack, P. D.; Randolph, S. J. Selected Area Deposition of PtCx Nanostructures: Implications for Functional Coatings of 3D Nanoarchitectures. *ACS Appl. Nano Mater.* **2022**, *5* (8), 10890–10899. https://doi.org/10.1021/acsanm.2c02182.

(2)	Singh, U. B.; Pannu, C.; Agarwal, D. C.; Ojha, S.; Khan, S. A.; Ghosh, S.; Avasthi, D. K. Large Electronic Sputtering Yield of Nanodimensional Au Thin Films: Dominant Role of Thermal Conductivity and Electron Phonon Coupling Factor. *J. Appl. Phys.* **2017**, *121* (9), 095308. https://doi.org/10.1063/1.4977845.

(3)	Werner, W. S. M.; Glantschnig, K.; Ambrosch-Draxl, C. Optical Constants and Inelastic Electron-Scattering Data for 17 Elemental Metals. *J. Phys. Chem. Ref. Data* **2009**, *38* (4), 1013–1092. https://doi.org/10.1063/1.3243762.
